# Supplementary material for: Ranking of cell clusters in a single-cell RNA-sequencing analysis framework using prior knowledge
Source: PLoS Comput Biol. 2024 Apr 18;20(4):e1011550. doi: 10.1371/journal.pcbi.1011550 (PMC11060557; doi:10.1371/journal.pcbi.1011550)
Supplement: S1 Text. Table A — Table B. Reactome Pathways according to MalaCards for “lymphangioleiomyomatosis”. Table C. Wiki pathways according to MalaCards for “lymphangioleiomyomatosis”. Table D. KEGG and other pathways from MalaCards for “lymphangioleiomyomatosis”. Table E. Gene Ontology (Biological Processes) from MalaCards for “lymphangioleiomyomatosis”. Table F. Drugs extracted from MalaCards for alias terms “Autism Disorder and Autism”. Table G. Reactome pathways from MalaCards for alias terms “Autism Disorder and Autism”. Table H. Wiki pathways from MalaCards for alias terms “Autism Disorder and Autism”. Table I. KEGG and other pathways from MalaCards for alias terms “Autism Disorder and Autism”. Table J. Gene Ontology (Biological Processes) from MalaCards for alias terms “Autism Disorder and Autism”. Table K. Drugs extracted from MalaCards for “COVID-19”. Table L. Reactome pathways from MalaCards for alias terms “COVID-19”. Table M. Wiki pathways from MalaCards for alias terms “COVID-19”. Table N. KEGG and other pathways from MalaCards for alias terms “COVID-19”. Table O. Gene Ontology (Biological Processes) from MalaCards for alias terms “COVID-19”. Fig A. Pathway enrichment analysis for LAM smooth muscle cell types. Fig B. Pathway enrichment analysis for LAM lung capillary aerocyte cell types. Fig C. Drug repurposing via enrichR for LAM smooth muscle cell types. Fig D. Pathway enrichment analysis for ASD IN-VIP cell types. Fig E. Pathway enrichment analysis for ASD endothelial cell types. Fig F. Drug repurposing via enrichR for ASD IN-VIP cell types. Fig G. Drug repurposing via enrichR for ASD endothelial cell types. Fig H. Pathway enrichment analysis for COVID-19 brain macrophage cell types. Fig I. Pathway enrichment analysis for COVID-19 brain astrocyte cell types. Fig J. Pathway enrichment analysis for COVID-19 brain excitatory neuron cell types. Fig K. Drug repurposing via enrichR for COVID-19 brain excitatory neuron cell types. (DOCX) [file pcbi.1011550.s001.docx]

**Supplementary Material**

**Ranking of cell clusters in a single-cell RNA-sequencing analysis framework using prior knowledge**

**Table A. Drugs extracted from MalaCards for “lymphangioleiomyomatosis”**

| Sirolimus |
| --- |
| Doxycycline |
| Everolimus |
| Simvastatin |
| Octreotide |
| Resveratrol |
| Celecoxib |
| Histamine |
| Progesterone |
| Nintedanib |
| Loratadine |
| Somatostatin |
| Letrozole |
| Saracatinib |
| Hydroxychloroquine |
| Salbutamol |
| Choline |
| Imatinib Mesylate |
| Histamine phosphate |
| Clotrimazole |
| Miconazole |
| Cetuximab |
| Bevacizumab |
| Valproic acid |
| L-Glutamine |
| Chondroitin |

**Table B. Reactome Pathways according to MalaCards for “lymphangioleiomyomatosis”**

| Signal Transduction |
| --- |
| Estrogen-stimulated signaling through PRKCZ |
| Stimulation of the cell death response by PAK-2p34 |
| Signaling by Receptor Tyrosine Kinases |
| Interferon alpha/beta signaling |
| Regulation of IFNA/IFNB signaling |
| Regulation of Insulin-like Growth Factor (IGF) transport and uptake by Insulin-like Growth Factor Binding Proteins (IGFBPs) |
| Post-translational protein phosphorylation |
| Extra-nuclear estrogen signaling |
| MTOR signalling |
| Energy dependent regulation of mTOR by LKB1-AMPK |
| Inhibition of TSC complex formation by PKB |
| mTORC1-mediated signalling |
| TP53 Regulates Metabolic Genes |
| VEGF binds to VEGFR leading to receptor dimerization |
| VEGF ligand-receptor interactions |

**Table C. Wiki pathways according to MalaCards for “lymphangioleiomyomatosis”**

| EGFR tyrosine kinase inhibitor resistance |
| --- |
| ErbB signaling pathway |
| Glioblastoma signaling pathways |
| TCA cycle nutrient use and invasiveness of ovarian cancer |
| Apoptosis |
| Apoptosis modulation by HSP70 |
| TNF-related weak inducer of apoptosis (TWEAK) signaling pathway |
| Butyrate-induced histone acetylation |
| Translation inhibitors in chronically activated PDGFRA cells |
| Endometrial cancer |
| Chromosomal and microsatellite instability in colorectal cancer |
| Melanoma |
| Non-small cell lung cancer |
| Pancreatic adenocarcinoma pathway |
| Overview of interferons-mediated signaling pathway |
| Type III interferon signaling |
| PI3K-Akt signaling pathway |
| Focal adhesion: PI3K-Akt-mTOR-signaling pathway |
| Angiopoietin-like protein 8 regulatory pathway |
| Insulin signaling |
| Liver X receptor pathway |
| Breast cancer pathway |
| Embryonic stem cell pluripotency pathways |
| exRNA mechanism of action and biogenesis |
| Malignant pleural mesothelioma |
| Focal adhesion |
| Integrated breast cancer pathway |
| Target of rapamycin signaling |
| Fragile X syndrome |
| Pilocytic astrocytoma |
| AMP-activated protein kinase signaling |
| Leptin and adiponectin |
| Thermogenesis |
| Senescence and autophagy in cancer |
| CCL18 signaling pathway |
| Clear cell renal cell carcinoma pathways |
| Thyroid hormones production and peripheral downstream signaling effects |
| Clock-controlled autophagy in bone metabolism |
| Head and neck squamous cell carcinoma |
| BDNF-TrkB signaling |
| Synaptic signaling pathways associated with autism spectrum disorder |
| MECP2 and associated Rett syndrome |
| Thyroid stimulating hormone (TSH) signaling pathway |
| Lung fibrosis |
| DYRK1A |
| Factors and pathways affecting insulin-like growth factor (IGF1)-Akt signaling |
| PI3K/AKT/mTOR - vitamin D3 signaling |
| Insulin signaling in adipocytes (normal condition) |
| IL-4 signaling pathway |
| Insulin signaling in adipocytes (diabetic condition) |
| Genes targeted by miRNAs in adipocytes |
| Mammary gland development pathway - Puberty (Stage 2 of 4) |
| Caloric restriction and aging |
| Somatroph axis (GH) and its relationship to dietary restriction and aging |

**Table D. KEGG and other pathways from MalaCards for “lymphangioleiomyomatosis”**

| ERK Signaling |
| --- |
| ILK Signaling |
| MAPK Signaling |
| Molecular Mechanisms of Cancer |
| GPCR Pathway |
| Breast Cancer Regulation by Stathmin1 |
| Estrogen Pathway |
| NFAT in Immune Response |
| P2Y Receptor Signaling |
| Pancreatic Adenocarcinoma |
| Paxillin Interactions |
| TGF-Beta Pathway |
| JAK-STAT Pathway |
| JNK Pathway |
| MAPK Family Pathway |
| Regulation of eIF4 and p70S6K |
| Prolactin Signaling |
| Development Prolactin receptor signaling |
| EGFR tyrosine kinase inhibitor resistance |
| ErbB signaling pathway |
| ErbB2-ErbB3 Heterodimers |
| Estrogen-stimulated signaling through PRKCZ |
| Glioblastoma signaling pathways |
| Growth Hormone Signaling |
| HMGB1 Pathway |
| IL-2 Pathway |
| LPS Stimulated MAPK Signaling |
| PACAP Signaling |
| TCA cycle nutrient use and invasiveness of ovarian cancer |
| Thrombopoietin Pathway |
| TREM1 Pathway |
| UVA-Induced MAPK Signaling |
| UVB-Induced MAPK Signaling |
| Akt Signaling |
| p38 Signaling |
| Nanog in Mammalian ESC Pluripotency |
| 14-3-3 Induced Intracellular Signaling |
| eNOS Signaling |
| Phospholipase-C Pathway |
| Apoptosis |
| Apoptosis modulation by HSP70 |
| Apoptosis through Death Receptors |
| Apoptotic Pathways Triggered By HIV1 |
| Ceramide signaling pathway |
| DR3 Signaling |
| GITR Pathway |
| Stimulation of the cell death response by PAK-2p34 |
| TNF-related weak inducer of apoptosis (TWEAK) signaling pathway |
| TRAIL Pathway |
| NFAT and Cardiac Hypertrophy |
| IGF1R Signaling |
| p70S6K Signaling |
| mTOR Pathway |
| Butyrate-induced histone acetylation |
| Cell adhesion PLAU signaling |
| Development CNTF receptor signaling |
| Development Growth hormone signaling via PI3K/AKT and MAPK cascades |
| Regulation of lipid metabolism Insulin signaling-generic cascades |
| Transcription Receptor-mediated HIF regulation |
| Translation Regulation of EIF2 activity |
| Pancreatic adenocarcinoma pathway |
| Overview of interferons-mediated signaling pathway |
| all-trans-Retinoic Acid Mediated Apoptosis |
| Cytokine Network |
| Interferon alpha/beta signaling |
| Development Dopamine D2 receptor transactivation of EGFR |
| Chemotaxis CXCR4 signaling pathway |
| G-protein signaling RAC1 in cellular process |
| CDC42 signaling events |
| Cytoskeleton remodeling CDC42 in cellular processes |
| MAPK Signaling: Mitogens |
| AMPK Enzyme Complex Pathway |
| mTOR Signaling |
| Transcription Androgen Receptor nuclear signaling |
| AMP-activated protein kinase signaling |
| Thermogenesis |
| TGF-beta Signaling Pathways |
| Interferon Pathway |
| G-protein signaling_RhoA regulation pathway |
| Cell adhesion_ECM remodeling |
| Regulation of Telomerase |
| mTOR signaling pathway |
| Development IGF-1 receptor signaling |
| Immune response IL-4 signaling pathway |
| LKB1 signaling events |
| Plasma membrane estrogen receptor signaling |
| G-protein signaling_Rap2B regulation pathway |
| p38 signaling mediated by MAPKAP kinases |
| Sirolimus Pathway, Pharmacokinetics/Pharmacodynamics |

**Table E. Gene Ontology (Biological Processes) from MalaCards for “lymphangioleiomyomatosis”**

| positive regulation of cell population proliferation |
| --- |
| positive regulation of MAPK cascade |
| response to hypoxia |
| aging |
| response to estrogen |
| protein kinase B signaling |
| response to heat |
| positive regulation of cell division |
| positive chemotaxis |
| cellular response to estradiol stimulus |
| ovulation from ovarian follicle |
| regulation of insulin-like growth factor receptor signaling pathway |
| phosphatidylinositol-mediated signaling |
| intramembranous ossification |
| positive regulation of activated T cell proliferation |
| positive regulation of mast cell chemotaxis |
| response to nutrient levels |
| positive regulation of smooth muscle cell proliferation |
| positive regulation of skeletal muscle tissue growth |
| insulin-like growth factor receptor signaling pathway |
| intracellular steroid hormone receptor signaling pathway |
| TOR signaling |
| response to insulin |

**Table F. Drugs extracted from MalaCards for alias terms “Autism Disorder and Autism”**

| Risperidone |
| --- |
| Oxytocin |
| Aripiprazole |
| Atomoxetine |
| Methylphenidate |
| Memantine |
| Dopamine |
| Serotonin |
| Bumetanide |
| Choline |
| Olanzapine |
| Valproic acid |
| Fluoxetine |
| Buspirone |
| Calcium |
| Sertraline |
| Sulforaphane |
| Melatonin |
| Norepinephrine |
| Insulin |
| Cannabidiol |
| Guanfacine |
| Histamine |
| Secretin |
| Galantamine |
| Iron |
| Acetylcysteine |
| Naltrexone |
| Everolimus |
| Arginine |
| Metformin |
| Dexmedetomidine |
| Cycloserine |
| Copper |
| Propranolol |
| Gabapentin |
| Minocycline |
| Mirtazapine |
| Ketamine |
| Cariprazine |
| Cysteine |
| Ziprasidone |
| Fluvoxamine |
| Creatine |
| Acetylcholine |
| Donepezil |
| Pioglitazone |
| Vitamin A |
| Acetaminophen |
| Riluzole |
| Citalopram |
| Prednisolone |
| Cobalamin |
| Tryptophan |
| Haloperidol |
| Folic acid |
| Baclofen |
| Sirolimus |
| Luteolin |
| Glycine |
| Iodine |
| Midazolam |
| Nicotine |
| Cholecalciferol |
| Clozapine |
| Acamprosate |
| Glutamic acid |
| Biotin |
| Propofol |
| Quinidine |
| Fluconazole |
| Selenium |
| Quercetin |
| Oxcarbazepine |
| Tianeptine |
| Curcumin |
| Taurine |
| Pyridoxine |
| Lamotrigine |
| Niacin |
| Spironolactone |
| Piperacillin |
| Carbamazepine |
| Nicotinamide |
| Psilocybin |
| Carbon monoxide |
| Lithium carbonate |
| Sodium Benzoate |
| Arachidonic Acid |
| Silicon |
| Milnacipran |
| Inositol |
| Pimavanserin |
| Lisdexamfetamine Dimesylate |
| Bisacodyl |
| Vancomycin |
| Cyanocobalamin |
| Hydroxocobalamin |
| Citric acid |
| Mecobalamin |
| Sodium citrate |
| Esomeprazole |
| Triamcinolone |
| Betahistine |
| Allopurinol |
| Amitriptyline |
| Methylprednisolone |
| Diphenhydramine |
| Zolpidem |
| Ascorbic acid |
| Ethanol |
| Brexpiprazole |
| Glycerin |
| Ergocalciferol |
| JNJ-42165279 |
| Triamcinolone hexacetonide |
| Suramin |
| Triamcinolone diacetate |
| Vitamin D2 |
| Dronabinol |
| Dimercaptosuccinic acid |
| Cathine |
| Guaifenesin |
| Clotrimazole |
| Hydrocortisone |
| Levoleucovorin |
| Miconazole |
| Magnesium sulfate |
| Lovastatin |
| Metyrosine |
| Mecasermin |
| Promethazine |
| Suvorexant |
| Benzocaine |
| Tannic acid |
| Prednisolone acetate |
| Dextroamphetamine |
| Histamine phosphate |
| Tocopherol |
| Paliperidone Palmitate |
| Tenamfetamine |
| Triamcinolone Acetonide |
| Lurasidone Hydrochloride |
| Oxycodone |
| Clemastine |
| Altretamine |
| Zinc cation |
| Lipoic acid |
| Lecithin |
| Carbamide peroxide |
| Lactulose |
| Calcium carbonate |
| Sodium fluoride |
| Lenalidomide |
| Acetylcarnitine |
| Magnesium citrate |
| Molybdenum |
| Verapamil |
| Ciprofloxacin |
| Cidofovir |
| Cefepime |
| Bosentan |
| Methylprednisolone hemisuccinate |
| Aminocaproic acid |
| Opium |
| Amikacin |
| Labetalol |
| Alfentanil |
| Rocuronium |
| Warfarin |
| Tranexamic acid |
| Nifedipine |
| Tazobactam |
| Nafcillin |
| Molindone |
| Vecuronium |
| Tobramycin |
| Pentobarbital |
| Fosfomycin |
| Nitrazepam |
| Aluminum hydroxide |
| Magnesium hydroxide |
| Coal tar |
| Hydrocortisone succinate |
| Hydrocortisone acetate |
| D-Tyrosine |
| Xylometazoline |
| Beta carotene |
| Hydroquinone |
| Butyric Acid |
| Caffeine |
| (3-Carboxy-2-(R)-Hydroxy-Propyl)-Trimethyl-Ammonium |
| Racemethionine |
| Phenethyl Isothiocyanate |
| Quinidine gluconate |
| Pantothenic acid |
| Ubidecarenone |
| Pyridoxal |
| Sennosides |
| Picolinic acid |
| Cadexomer iodine |
| Dimethyl sulfoxide |
| Tocotrienol |
| Mecamylamine |
| Cod liver oil |
| Palmidrol |
| Sulfamethazine |
| Sevelamer |
| Prednisolone phosphate |
| Simethicone |
| Sodium oxybate |
| Silver sulfadiazine |
| Iron isomaltoside 1000 |
| L-Glutamine |
| Isotretinoin |
| DL-alpha-Tocopherol |
| Trichostatin A |
| Prednisolone hemisuccinate |
| Dexetimide |
| Haloperidol decanoate |
| Methylprednisolone Acetate |
| TEMPO |
| D-Leucine |

**Table G. Reactome pathways from MalaCards for alias terms “Autism Disorder and Autism”**

| Defective SLC9A9 causes autism 16 (AUTS16) |
| --- |

**Table H. Wiki pathways from MalaCards for alias terms “Autism Disorder and Autism”**

| Pathways affected in adenoid cystic carcinoma |
| --- |
| Thyroid hormones production and peripheral downstream signaling effects |
| Rett syndrome causing genes |
| Dravet syndrome |
| Hematopoietic stem cell gene regulation by GABP alpha/beta complex |

**Table I. KEGG and other pathways from MalaCards for alias terms “Autism Disorder and Autism”**

| Neuroscience |
| --- |
| Effects of Botulinum toxin |

**Table J. Gene Ontology (Biological Processes) from MalaCards for alias terms “Autism Disorder and Autism”**

| social behavior |
| --- |
| positive regulation of excitatory postsynaptic potential |
| regulation of synaptic vesicle priming |
| long-term synaptic potentiation |
| presynaptic membrane assembly |
| membrane depolarization during action potential |
| neuronal action potential |
| maintenance of postsynaptic density structure |
| prepulse inhibition |

**Table K. Drugs extracted from MalaCards for “COVID-19”**

| Dexamethasone |
| --- |
| Angiotensin II |
| Testosterone |
| Fluvoxamine |
| Favipiravir |
| Darunavir |
| Ritonavir |
| Sofosbuvir |
| Chloroquine |
| Losartan |
| Colchicine |
| Bromhexine |
| Lopinavir |
| Infliximab |
| Bamlanivimab |
| Melatonin |
| Montelukast |
| Spironolactone |
| Rivaroxaban |
| Calcium |
| Nicotine |
| Nitazoxanide |
| Copper |
| Pirfenidone |
| Tofacitinib |
| Nafamostat |
| Iodine |
| Fluoxetine |
| Lenalidomide |
| Amantadine |
| Povidone-iodine |
| Atorvastatin |
| Tenofovir |
| Dapagliflozin |
| Apixaban |
| Nintedanib |
| Telmisartan |
| Methotrexate |
| Selenium |
| Cannabidiol |
| Amlodipine |
| Ursodeoxycholic acid |
| Zinc sulfate |
| Ramipril |
| Estradiol |
| Dopamine |
| Cholecalciferol |
| Lactoferrin |
| Indomethacin |
| Bismuth |
| Curcumin |
| Ofatumumab |
| Ciclesonide |
| Cobicistat |
| Ribavirin |
| Sevoflurane |
| Iron |
| Ginseng |
| Argatroban |
| Artesunate |
| Epigallocatechin |
| Levamisole |
| Fenofibrate |
| Resveratrol |
| Valsartan |
| Itraconazole |
| Calcifediol |
| Fluticasone |
| Rituximab |
| Interferon beta-1b |
| Thalidomide |
| Mavrilimumab |
| Nifedipine |
| Interferon beta-1a |
| Raloxifene |
| Sargramostim |
| Cysteamine |
| Abatacept |
| Glycine |
| Adenosine |
| Propofol |
| Protein C |
| Maraviroc |
| BCG vaccine |
| Clarithromycin |
| Ibuprofen |
| Ketotifen |
| Apremilast |
| Nicotinamide |
| Mefloquine |
| Candesartan |
| Serotonin |
| Bemiparin |
| Naproxen |
| Arginine |
| Progesterone |
| Diltiazem |
| Defibrotide |
| Pyronaridine |
| Nimodipine |
| Andrographolide |
| Colistin |
| Fisetin |
| Celecoxib |
| Lidocaine |
| Licorice |
| Dapsone |
| Artemether |
| Lysine |
| Luteolin |
| Papain |
| Moxifloxacin |
| Disulfiram |
| Eculizumab |
| Ravulizumab |
| Silibinin |
| Cysteine |
| Fentanyl |
| Theophylline |
| Sildenafil |
| Hesperidin |
| Brequinar |
| Omalizumab |
| Methylene blue |
| Ocrelizumab |
| Phenol |
| Methadone |
| Tetracycline |
| Siltuximab |
| Dutasteride |
| Mometasone furoate |
| Hydrogen Sulfide |
| Nivolumab |
| Buprenorphine |
| Epoprostenol |
| Thrombin |
| Protoporphyrin |
| Vortioxetine |
| Cianidanol |
| Bradykinin |
| Warfarin |
| Tamoxifen |
| Levofloxacin |
| Adrenomedullin |
| Zafirlukast |
| Cetylpyridinium |
| Folic acid |
| Cepharanthine |
| Natalizumab |
| Genistein |
| Epicatechin |
| Diazepam |
| bilirubin |
| Ondansetron |
| Calcitonin |
| Galidesivir |
| Aprotinin |
| Epigallocatechin gallate |
| Fenretinide |
| Metoprolol |
| Acetylcholine |
| Amiodarone |
| Ceftazidime |
| Cefepime |
| Probenecid |
| Mercaptopurine |
| Hyaluronic acid |
| Vedolizumab |
| Opium |
| Tryptophan |
| Azacitidine |
| TEMPO |
| Pembrolizumab |
| Garlic |
| Uric acid |
| Chlorine dioxide |
| Meropenem |
| Sertraline |
| Ustekinumab |
| Metronidazole |
| Ciprofloxacin |
| Creatine |
| Rosemary |
| Calcitonin gene-related peptide |
| Taxifolin |
| Deferoxamine |
| Pioglitazone |
| Cyproheptadine |
| Ledipasvir |
| Clopidogrel |
| Hydrogen peroxide |
| Cefditoren |
| Midazolam |
| Fondaparinux |
| Ketamine |
| Glucosamine |
| Bentonite |
| Isotretinoin |
| Acalabrutinib |
| Empagliflozin |
| Dextroamphetamine |
| Morphine |
| Bismuth subsalicylate |
| Loperamide |
| Tetrandrine |
| Oseltamivir |
| Ticagrelor |
| Dipyridamole |
| Vitamin D2 |
| Bevacizumab |
| Enalaprilat |
| Enalapril |
| Canagliflozin |
| Verapamil |
| Ertugliflozin |
| Dexamethasone acetate |
| Icatibant |
| Peppermint |
| Ergocalciferol |
| Mycophenolic acid |
| Valproic acid |
| Chlorothiazide |
| Captopril |
| Metolazone |
| Hydrochlorothiazide |
| Leronlimab |
| Lisinopril |
| Chlorthalidone |
| Chlorpromazine |
| Indapamide |
| Quinapril |
| Bendroflumethiazide |
| Benazepril |
| Trandolapril |
| Quinidine |
| Primaquine |
| Nicardipine |
| Nitrendipine |
| Felodipine |
| Evolocumab |
| Chlorpheniramine |
| Fosinopril |
| Perindopril |
| Edoxaban |
| Furosemide |
| Reviparin |
| Coal tar |
| Ambrisentan |
| Isoflurane |
| Cenicriviroc |
| Ofloxacin |
| Tenecteplase |
| Ibudilast |
| Meplazumab |
| Chlorhexidine |
| Stanolone |
| Maleic acid |
| Nebivolol |
| Nicorandil |
| Angiotensinogen |
| Albendazole |
| Sodium Hypochlorite |
| Loratadine |
| Eicosapentaenoic acid ethyl ester |
| Sorbitol |
| Quetiapine Fumarate |
| Palmidrol |
| Lansoprazole |
| Isoquercetin |
| Cod liver oil |
| Histidine |
| D-Proline |
| Candesartan cilexetil |
| Irbesartan |
| Olmesartan |
| Imatinib Mesylate |
| Crofelemer |
| Remestemcel-L |
| Inosine pranobex |
| Ropivacaine |
| Silver nitrate |
| Racepinephrine |
| Fludrocortisone |
| Cyanocobalamin |
| Sodium fluoride |
| Etoposide |
| Ascorbic acid |
| Pyrazinamide |
| Glycerin |
| Thymalfasin |
| Betadex |
| Titanium dioxide |
| Phentolamine |
| Ifenprodil |
| Clevudine |
| Betamethasone |
| Benzyl alcohol |
| Aldesleukin |
| Ixekizumab |
| Melphalan |
| Trimetazidine |
| Diphenhydramine |
| Iloprost |
| Tafenoquine |
| Racivir |
| Tirofiban |
| Pancrelipase |
| Regadenoson |
| Lumefantrine |
| Plerixafor |
| Decitabine |
| Fennel |
| Ginger |
| Roxithromycin |
| Ceftaroline fosamil |
| Eleuthero |
| Aluminum sulfate |
| Almitrine |
| Levoleucovorin |
| Ozanimod |
| Indian frankincense |
| Aprepitant |
| Prazosin |
| Niacin |
| Riboflavin |
| Silver sulfadiazine |
| Hydroxocobalamin |
| Avdoralimab |
| Mecobalamin |
| Gimsilumab |
| Tocopherol |
| Sulfamethoxazole |
| Trimethoprim |
| Zinc cation |
| Ebselen |
| Dexlansoprazole |
| Amoxicillin |
| Piperacillin |
| Eprosartan |
| Eltanolone |
| Sage |
| Phylloquinone |
| Xenon |
| Psilocybin |
| Salicylic acid |
| Bisoprolol |
| Carbamazepine |
| Topotecan |
| Gabapentin |
| Azilsartan medoxomil |
| Insulin aspart |
| Glutamic acid |
| Alfacalcidol |
| Elsulfavirine |
| Lecithin |
| L-Glutamine |
| Racemethionine |
| Menadione |
| Tramadol |
| (3-Carboxy-2-(R)-Hydroxy-Propyl)-Trimethyl-Ammonium |
| Bivalirudin |
| Modafinil |
| Nadroparin |
| Methyltestosterone |
| Pyrazole |
| Estrone |
| Turmeric |
| Cerebrolysin |
| Ramatroban |
| Corticosterone |
| Molgramostim |
| Gabexate |
| Bucillamine |
| Cobalamin |
| Tempol |
| Salmon calcitonin |
| Pyridostigmine Bromide |
| Nitroglycerin |
| Neurokinin A |
| Prasugrel Hydrochloride |
| Rosuvastatin Calcium |
| Cromolyn Sodium |
| Sitagliptin Phosphate |
| Clindamycin |
| Naphthoquinone |
| Triamcinolone |
| Alprostadil |
| Clofazimine |
| Zanubrutinib |
| Benzylpenicillin |
| Sorafenib |
| Petrolatum |
| Amodiaquine |
| Cimetidine |
| Peppermint oil |
| Mupirocin |
| Mepivacaine |
| Fenofibric acid |
| Cathine |
| Guaifenesin |
| Carbamide peroxide |
| Caffeine |
| Metamizole |
| Dimethyl sulfoxide |
| Calcium carbonate |
| Calcitriol |
| Flavin mononucleotide |
| Magnesium sulfate |
| Glycopyrronium |
| Echinacea |
| Canrenoic acid |
| Altretamine |
| Phenformin |
| Basiliximab |
| Selenious acid |
| Minocycline |
| Alitretinoin |
| Polyestradiol phosphate |
| Wormwood |
| Povidone K30 |
| Magnesium citrate |
| Buserelin |
| Ubidecarenone |
| D-Tyrosine |
| D-Threonine |
| Thiamine |
| Pantothenic acid |
| Aspartic acid |
| Manganese |
| Trabedersen |
| Stevioside |
| Abivertinib |
| Tezosentan |
| Astegolimab |
| Glenzocimab |
| Nangibotide |
| Dalcetrapib |
| Dapansutrile |
| Apigenin |
| 3,4-Dihydroxycinnamic Acid |
| Canrenone |
| Perflubron |
| Zansecimab |
| Picolinic acid |
| Varespladib methyl |
| Avasopasem manganese |
| Dactolisib |
| Triamcinolone hexacetonide |
| Triamcinolone diacetate |
| Triamcinolone Acetonide |
| Hydroxyitraconazole |
| Sodium thiosulfate |
| Beclomethasone |
| Vitamin B2 |
| Etoposide phosphate |
| D-Alanine |
| Promethazine |
| Ranitidine |
| Menthol |
| Methyl salicylate |
| Diclofenac |
| Bupivacaine |
| Salbutamol |
| Framycetin |
| Bacitracin |
| Aluminium phosphate |
| Rifampicin |
| Tin protoporphyrin IX |
| Camptothecin |
| Cloricromen |
| Trichostatin A |
| Ranitidine bismuth citrate |
| Salicylates |
| Quinidine gluconate |
| Lithium carbonate |
| Metoclopramide |
| Bromazepam |
| Acetylcarnitine |
| Trastuzumab |
| Pertuzumab |
| Papaverine |
| Parathyroid hormone |
| Clonidine |
| Dabigatran |
| Xylometazoline |
| Glycyrrhizic acid |
| Moclobemide |
| Silicon |
| Testosterone undecanoate |
| Testosterone enanthate |
| Racephedrine |
| Fexofenadine |
| Cangrelor |
| Acenocoumarol |
| Carbimazole |
| Erenumab |
| Simethicone |
| Timolol |
| Minoxidil |
| Eplerenone |
| Bumetanide |
| Amiloride |
| Betaxolol |
| Hydralazine |
| Atenolol |
| Methyldopa |
| Isradipine |
| Terazosin |
| Acebutolol |
| Propranolol |
| Torasemide |
| Doxazosin |
| Penbutolol |
| Reserpine |
| Nisoldipine |
| Triamterene |
| Nadolol |
| Moexipril |
| Pindolol |
| Guanabenz |
| Etacrynic acid |
| Pregabalin |
| Pholcodine |
| Cefotaxime |
| Nitrofurantoin |
| Nutmeg |
| Dalfampridine |
| Carvedilol |
| Posaconazole |
| Clobazam |
| Cefdinir |
| Bosentan |
| Aminocaproic acid |
| Labetalol |
| Milrinone |
| Terbutaline |
| Sevelamer |
| Risperidone |
| Voriconazole |
| Oxycodone |
| Nalbuphine |
| Zolpidem |
| Guanfacine |
| Fosfomycin |
| Naloxone |
| Alemtuzumab |
| Teriflunomide |
| Nitroprusside |
| Atezolizumab |
| Docetaxel |
| Ezetimibe |
| Amikacin |
| Ethinylestradiol |
| Dimethyl fumarate |
| Carbon monoxide |
| Edetate calcium disodium anhydrous |
| Pentetic acid |
| Sulfamethazine |
| Rabeprazole |
| Nizatidine |
| Pantoprazole |
| Formaldehyde |
| Paroxetine |
| Venlafaxine |
| Certolizumab pegol |
| Mesalazine |
| Pimozide |
| Thyme |
| Polymyxin B |
| Meticillin |
| Dimercaprol |
| Esomeprazole |
| Esketamine |
| Tazobactam |
| Ertapenem |
| Cetrorelix |
| Clomifene |
| Dydrogesterone |
| St. John's Wort |
| DL-alpha-Tocopherol |
| Prasterone |
| Inulin |
| Beta carotene |
| D-Phenylalanine |
| Lipoic acid |
| Pyridoxine |
| 2,4-thiazolidinedione |
| Ferulic acid |
| Lobeline |
| Fenbendazole |
| Chitosan low molecular weight (20-200 mpa.s) |
| Tocotrienol |
| gamma-Tocopherol |
| Cadexomer iodine |
| Sivelestat |
| Xanthohumol |
| Enclomiphene |
| Oleuropein |
| Pyrithione zinc |
| Terephthalic acid |
| Boswellic acid |
| Estropipate |
| Brimonidine Tartrate |
| Nickel |
| abobotulinumtoxinA |
| Arginyl-glycyl-aspartic acid |
| beta-Endorphin |
| PK 11195 |
| Chalcone |
| Pyridoxal |
| D-Leucine |

**Table L. Reactome pathways from MalaCards for alias terms “COVID-19”**

| Innate Immune System |
| --- |
| Immune System |
| Neutrophil degranulation |
| Disease |
| Infectious disease |
| Signal Transduction |
| Cytokine Signaling in Immune system |
| Signaling by Interleukins |
| SARS-CoV-2 Infection |
| SARS-CoV Infections |
| SARS-CoV-2 activates/modulates innate and adaptive immune responses |
| SARS-CoV-2-host interactions |
| Interferon alpha/beta signaling |
| Regulation of IFNA/IFNB signaling |
| Interleukin-33 signaling |
| Interleukin-36 pathway |
| SARS-CoV-1-host interactions |
| SARS-CoV-1 activates/modulates innate immune responses |
| SARS-CoV-1 Infection |
| SARS-CoV-1 targets host intracellular signalling and regulatory pathways |
| Cytosolic sensors of pathogen-associated DNA |
| DEx/H-box helicases activate type I IFN and inflammatory cytokines production |
| IkBA variant leads to EDA-ID |
| IRF3 mediated activation of type 1 IFN |
| IRF3-mediated induction of type I IFN |
| LRR FLII-interacting protein 1 (LRRFIP1) activates type I IFN production |
| Regulation by TREX1 |
| Regulation of innate immune responses to cytosolic DNA |
| RIP-mediated NFkB activation via ZBP1 |
| STAT6-mediated induction of chemokines |
| STING mediated induction of host immune responses |
| ZBP1(DAI) mediated induction of type I IFNs |
| Early SARS-CoV-2 Infection Events |
| Attachment and Entry |
| Attachment and Entry |
| Defective MOGS causes CDG-2b |
| Maturation of spike protein |
| Translation of Replicase and Assembly of the Replication Transcription Complex |
| Translation of Replicase and Assembly of the Replication Transcription Complex |
| Translation of Structural Proteins |
| DDX58/IFIH1-mediated induction of interferon-alpha/beta |
| Negative regulators of DDX58/IFIH1 signaling |
| NF-kB activation through FADD/RIP-1 pathway mediated by caspase-8 and -10 |
| TRAF3-dependent IRF activation pathway |
| TRAF6 mediated IRF7 activation |
| Interleukin-4 and Interleukin-13 signaling |
| Inhibition of PKR |
| Interleukin-10 signaling |

**Table M. Wiki pathways from MalaCards for alias terms “COVID-19”**

| Overview of proinflammatory and profibrotic mediators |
| --- |
| Toll-like receptor signaling pathway |
| Hepatitis B infection |
| Measles virus infection |
| Regulation of toll-like receptor signaling pathway |
| Malignant pleural mesothelioma |
| Overview of interferons-mediated signaling pathway |
| Type III interferon signaling |
| Interleukin-1 (IL-1) structural pathway |
| Nanomaterial-induced inflammasome activation |
| TLR4 signaling and tolerance |
| Cytosolic DNA-sensing pathway |
| Selenium micronutrient network |
| SARS-CoV-1 Infection |
| SARS-CoV-2 Infection |
| Novel intracellular components of RIG-I-like receptor pathway |
| SARS-CoV-2 B.1.1.7 variant antagonises innate immune activation |
| Altered glycosylation of MUC1 in tumor microenvironment |
| Signal transduction through IL1R |
| IL-17 signaling pathway |
| Sudden infant death syndrome (SIDS) susceptibility pathways |
| Host-pathogen interaction of human coronaviruses - interferon induction |
| SARS coronavirus and innate immunity |
| Type I interferon induction and signaling during SARS-CoV-2 infection |
| Spinal cord injury |
| Allograft rejection |
| CCL18 signaling pathway |
| Network map of SARS-CoV-2 signaling pathway |
| Pathogenesis of SARS-CoV-2 mediated by nsp9-nsp10 complex |
| Canonical NF-kB pathway |
| STING pathway in Kawasaki-like disease and COVID-19 |
| Extrafollicular B cell activation by SARS-CoV-2 |
| Burn wound healing |
| Th17 cell differentiation pathway |
| Photodynamic therapy-induced NF-kB survival signaling |
| Lung fibrosis |
| miRNAs involvement in the immune response in sepsis |
| Inflammatory bowel disease signaling |
| Ulcerative colitis signaling |
| Non-genomic actions of 1,25 dihydroxyvitamin D3 |
| SARS-CoV-2 innate immunity evasion and cell-specific immune response |
| Interactions between immune cells and microRNAs in tumor microenvironment |
| Nonalcoholic fatty liver disease |
| Fibrin complement receptor 3 signaling pathway |
| Mitochondrial immune response to SARS-CoV-2 |
| SARS-CoV-2 mitochondrial chronic oxidative stress and endothelial dysfunction |
| Immune infiltration in pancreatic cancer |
| Development and heterogeneity of the ILC family |
| Cytokines and inflammatory response |
| Cell interactions of the pancreatic cancer microenvironment |
| IL1 and megakaryocytes in obesity |
| Antiviral and anti-inflammatory effects of Nrf2 on SARS-CoV-2 pathway |
| LDL- influence on CD14 and TLR4 |
| Prostaglandin signaling |
| Overview of nanoparticle effects |
| Activation of NLRP3 inflammasome by SARS-CoV-2 |
| COVID-19 adverse outcome pathway |
| Cells and molecules involved in local acute inflammatory response |
| SARS-CoV-2 altering angiogenesis via NRP1 |
| SARS-CoV-2 and COVID-19 pathway |

**Table N. KEGG and other pathways from MalaCards for alias terms “COVID-19”**

| MIF Mediated Glucocorticoid Regulation |
| --- |
| all-trans-Retinoic Acid Signaling in Brain |
| Endothelin-1 Signaling Pathway |
| Glucocorticoid Receptor Signaling |
| IL-6 Pathway |
| MIF Regulation of Innate Immune Cells |
| Overview of proinflammatory and profibrotic mediators |
| PEDF Induced Signaling |
| PGC1Alpha Pathway |
| RAR-Gamma-RXR-Alpha Degradation |
| Akt Signaling |
| p38 Signaling |
| PAK Pathway |
| Antioxidant Action of Vitamin-C |
| NF-KappaB Family Pathway |
| Overview of interferons-mediated signaling pathway |
| all-trans-Retinoic Acid Mediated Apoptosis |
| Cytokine Network |
| Interferon alpha/beta signaling |
| IL-1 Family Signaling Pathways |
| IL1 signaling pathway |
| Interleukin-36 pathway |
| Mucin expression in CF via TLRs, EGFR signaling pathways |
| NF-kappaB Signaling |
| Toll-Like receptor Signaling Pathways |
| Nanomaterial-induced inflammasome activation |
| TLR4 signaling and tolerance |
| DDX58/IFIH1-mediated induction of interferon-alpha/beta |
| Toll Comparative Pathway |
| IL-1 Pathway |
| Immune response MIF in innate immunity response |
| iNOS Signaling |
| Altered glycosylation of MUC1 in tumor microenvironment |
| IL-1 beta-dependent CFTR expression |
| IL1-mediated signaling events |
| Immune response Bacterial infections in normal airways |
| Immune response TLR signaling pathways |
| IL-17 Family Signaling Pathways |
| IL-17 signaling pathway |
| Colorectal Cancer Metastasis |
| MAPK Pathway |
| IL27-mediated signaling events |
| IL12 signaling pathway |
| Host-pathogen interaction of human coronaviruses - interferon induction |
| Th17 Differentiation |
| Cytokine production by Th17 cells in CF (Mouse model) |
| Dendritic Cells Developmental Lineage Pathway |
| Canonical NF-kB pathway |
| Innate Lymphoid Cells Differentiation |
| AP-1 transcription factor network |
| Photodynamic therapy-induced NF-kB survival signaling |
| Calcineurin-regulated NFAT-dependent transcription in lymphocytes |
| IL 10 signaling pathway |
| MSP-RON Signaling |
| Transcription_Role of VDR in regulation of genes involved in osteoporosis |
| IL-10 Pathway |
| Validated transcriptional targets of AP1 family members Fra1 and Fra2 |
| A-beta Uptake and Degradation |
| Cellular roles of Anthrax toxin |
| G-protein signaling_Rap2B regulation pathway |
| LDL Oxidation in Atherogenesis |
| IL-9 Signaling and its Primary Biological Effects in Different Immune Cell Types |
| Cells and molecules involved in local acute inflammatory response |

**Table O. Gene Ontology (Biological Processes) from MalaCards for alias terms “COVID-19”**

| signal transduction |
| --- |
| positive regulation of gene expression |
| positive regulation of DNA-binding transcription factor activity |
| viral entry into host cell |
| MAPK cascade |
| defense response to virus |
| neutrophil chemotaxis |
| defense response to Gram-positive bacterium |
| positive regulation of type II interferon production |
| positive regulation of NF-kappaB transcription factor activity |
| positive regulation of NIK/NF-kappaB signaling |
| positive regulation of interleukin-1 beta production |
| regulation of insulin secretion |
| positive regulation of inflammatory response |
| positive regulation of nitric oxide biosynthetic process |
| cellular response to virus |
| response to lipopolysaccharide |
| positive regulation of interferon-beta production |
| JNK cascade |
| positive regulation of interleukin-6 production |
| cellular response to organic cyclic compound |
| positive regulation of cytokine production involved in inflammatory response |
| I-kappaB kinase/NF-kappaB signaling |
| negative regulation of neurogenesis |
| lipopolysaccharide-mediated signaling pathway |
| response to bacterium |
| positive regulation of glial cell proliferation |
| positive regulation of membrane protein ectodomain proteolysis |
| activation of innate immune response |
| positive regulation of interleukin-8 production |
| regulation of inflammatory response |
| cellular response to interleukin- |
| positive regulation of oxidative stress-induced neuron death |
| regulation of systemic arterial blood pressure by renin-angiotensin |
| positive regulation of neuroinflammatory response |
| immune response |
| positive regulation of fever generation |
| receptor-mediated endocytosis of virus by host cell |
| defense response |
| viral life cycle |
| positive regulation of chemokine production |
| sequestering of triglyceride |
| angiogenesis involved in coronary vascular morphogenesis |
| positive regulation of calcidiol 1-monooxygenase activity |
| negative regulation of gap junction assembly |
| vascular endothelial growth factor production |
| cellular response to lipopolysaccharide |
| response to external stimulus |
| inflammatory response |

**Fig A. Pathway enrichment analysis for LAM smooth muscle cell types
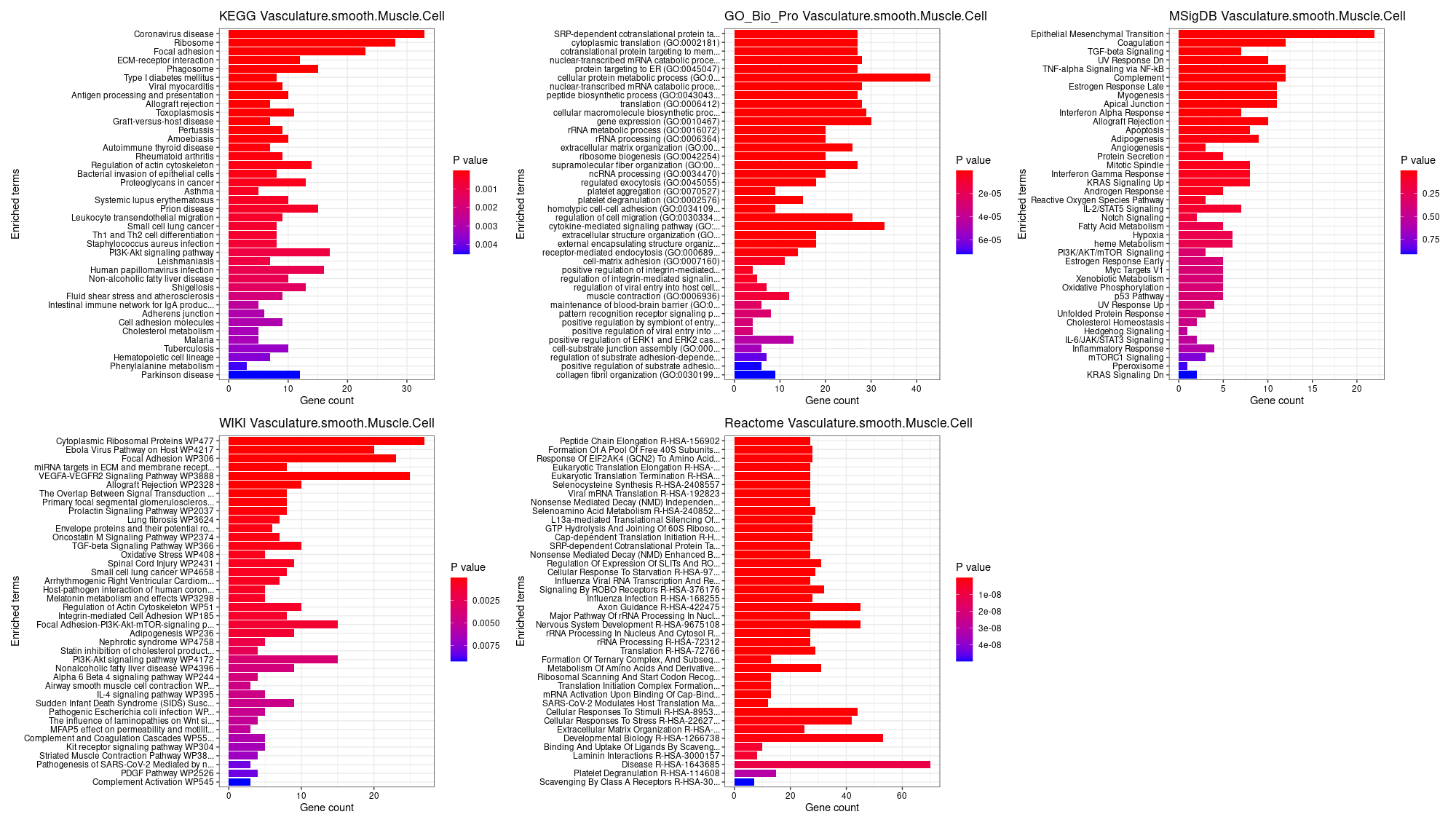
**

**Fig B. Pathway enrichment analysis for LAM lung capillary aerocyte cell types**

**
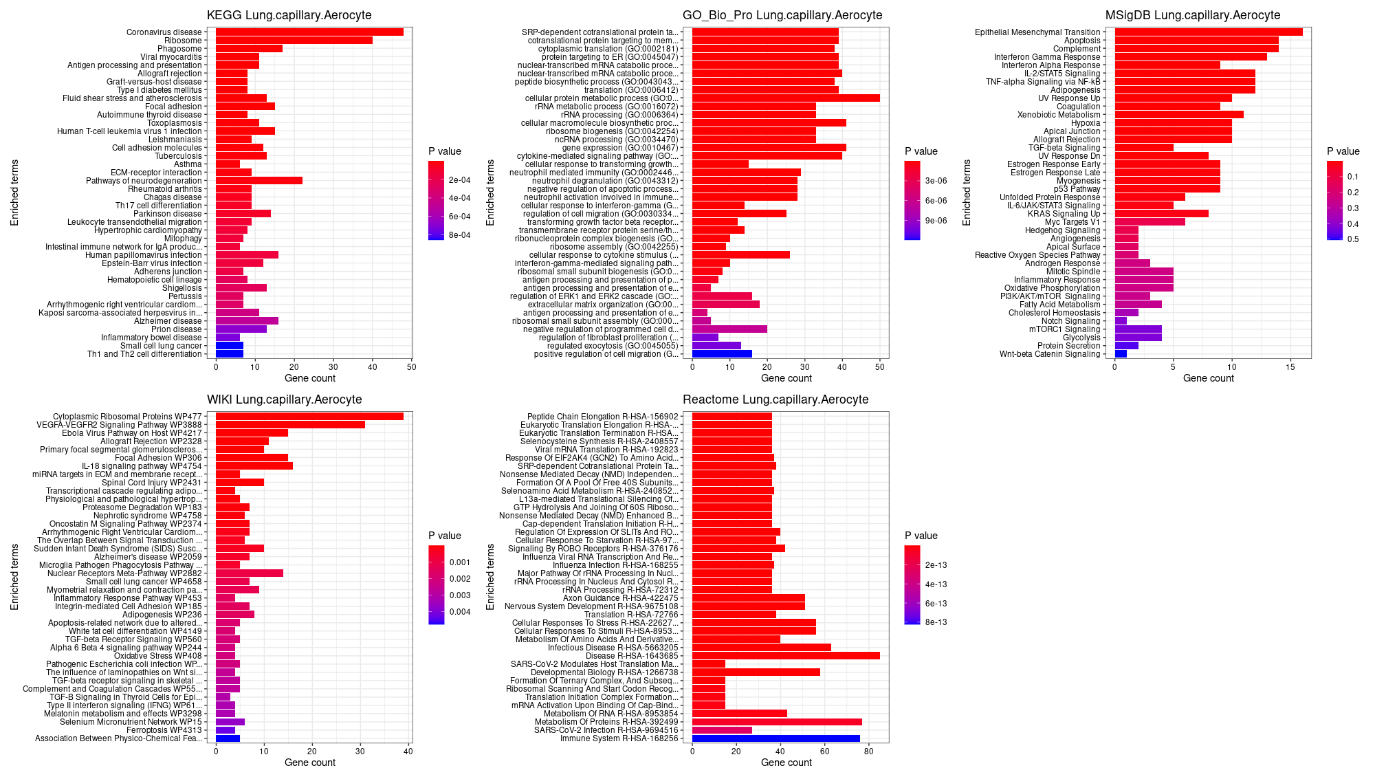
**

**Fig C. Drug repurposing via enrichR for LAM smooth muscle cell types**

**
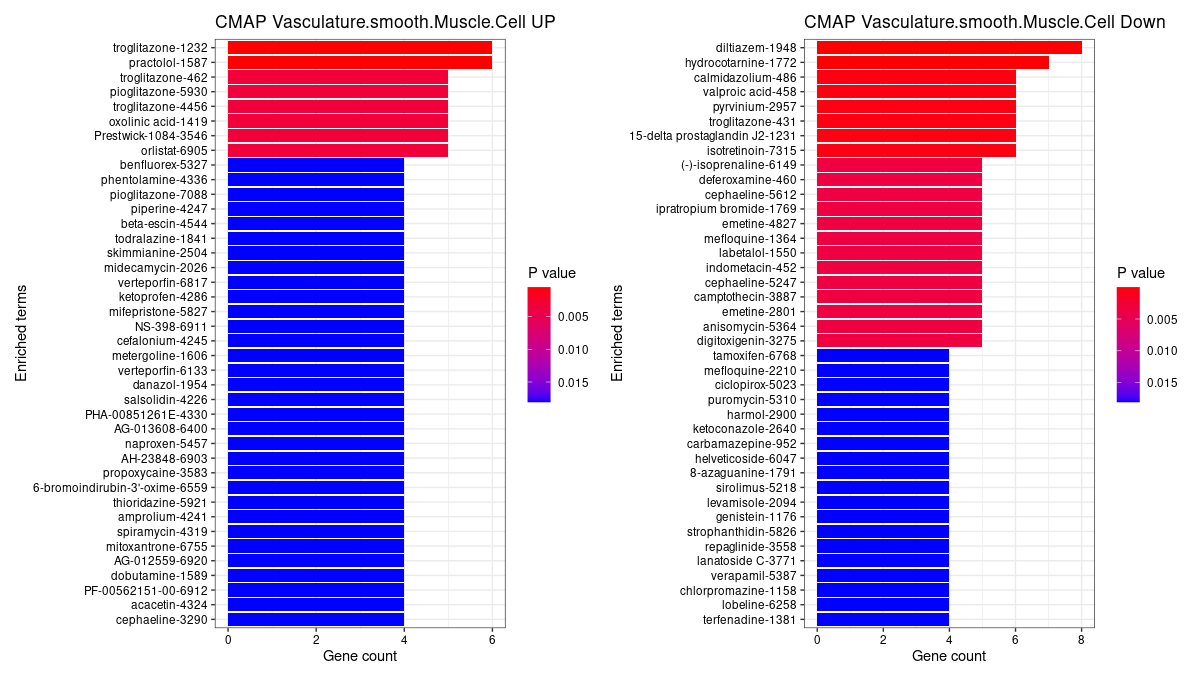
**

**
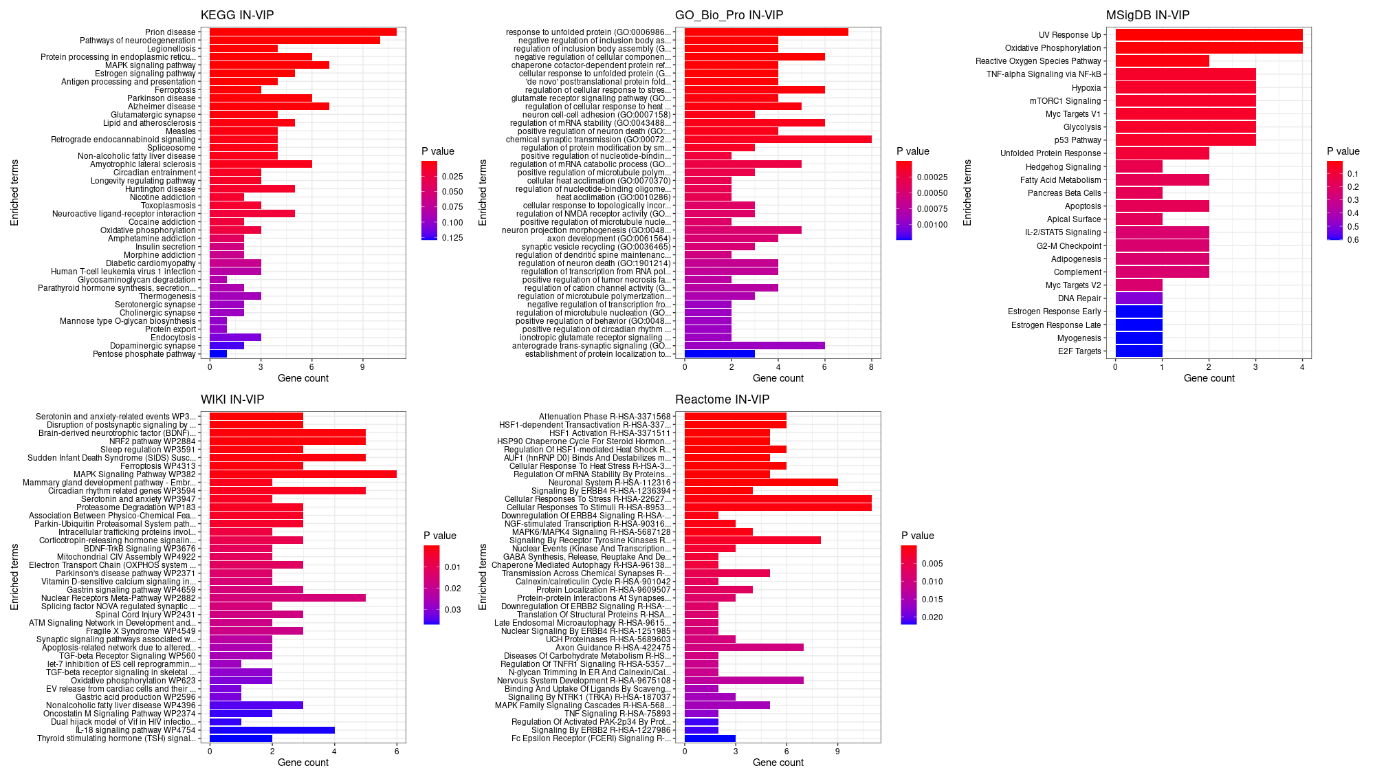
Fig D. Pathway enrichment analysis for ASD IN-VIP cell types**

**Fig E. Pathway enrichment analysis for ASD endothelial cell types**

**
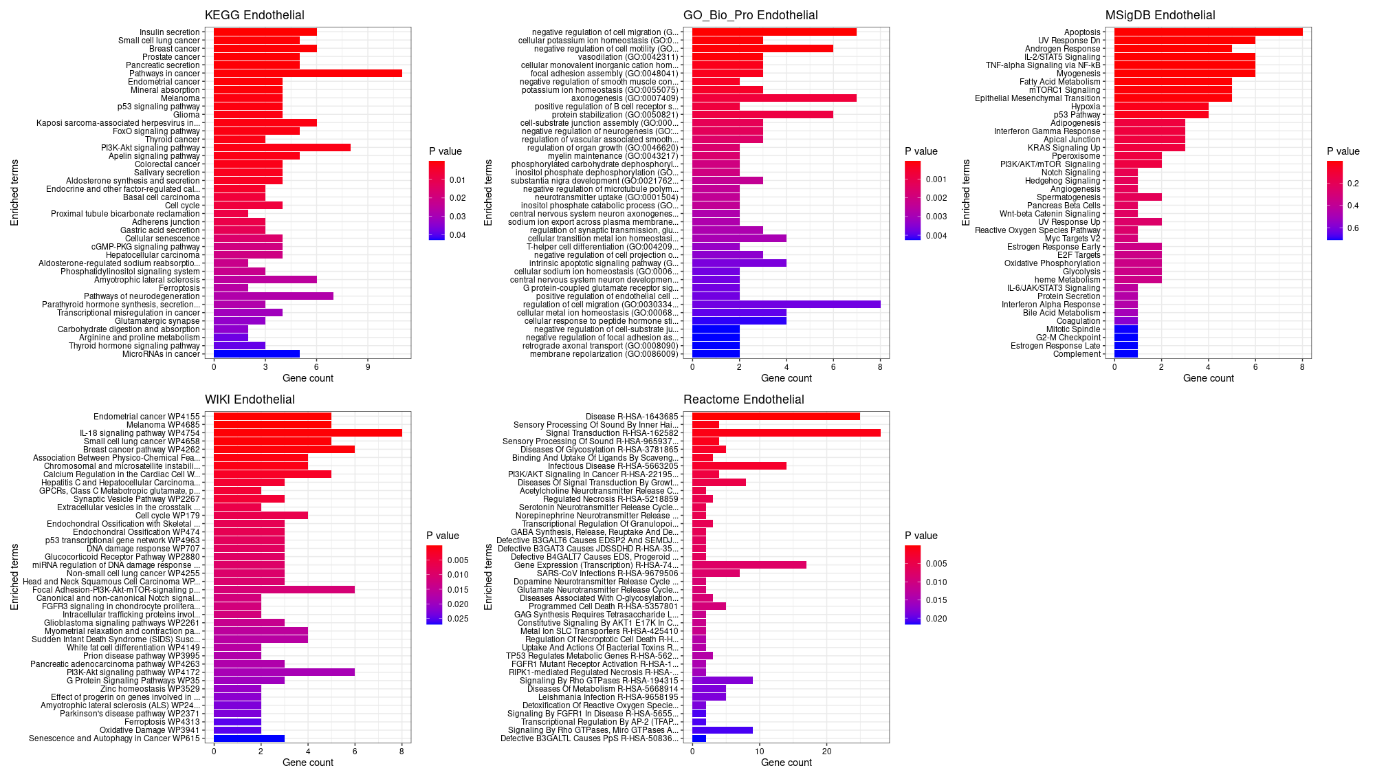
**

**Fig F. Drug repurposing via enrichR for ASD IN-VIP cell types**

**
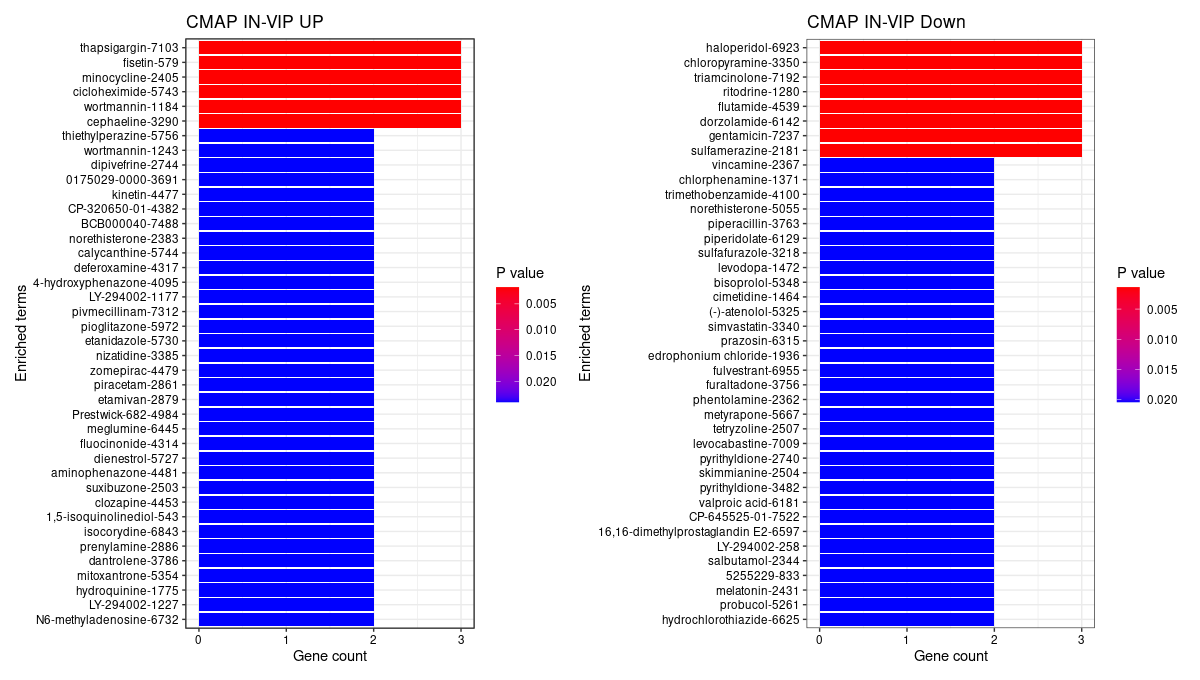
**

**Fig G. Drug repurposing via enrichR for ASD endothelial cell types**

**
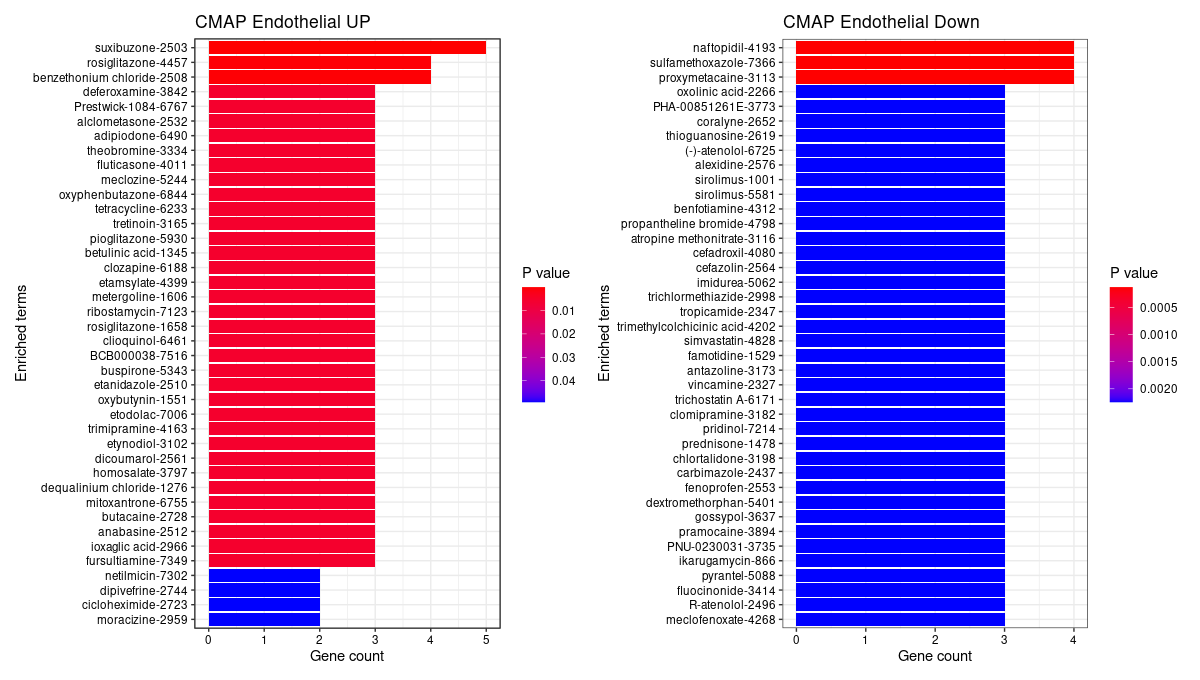
**

**
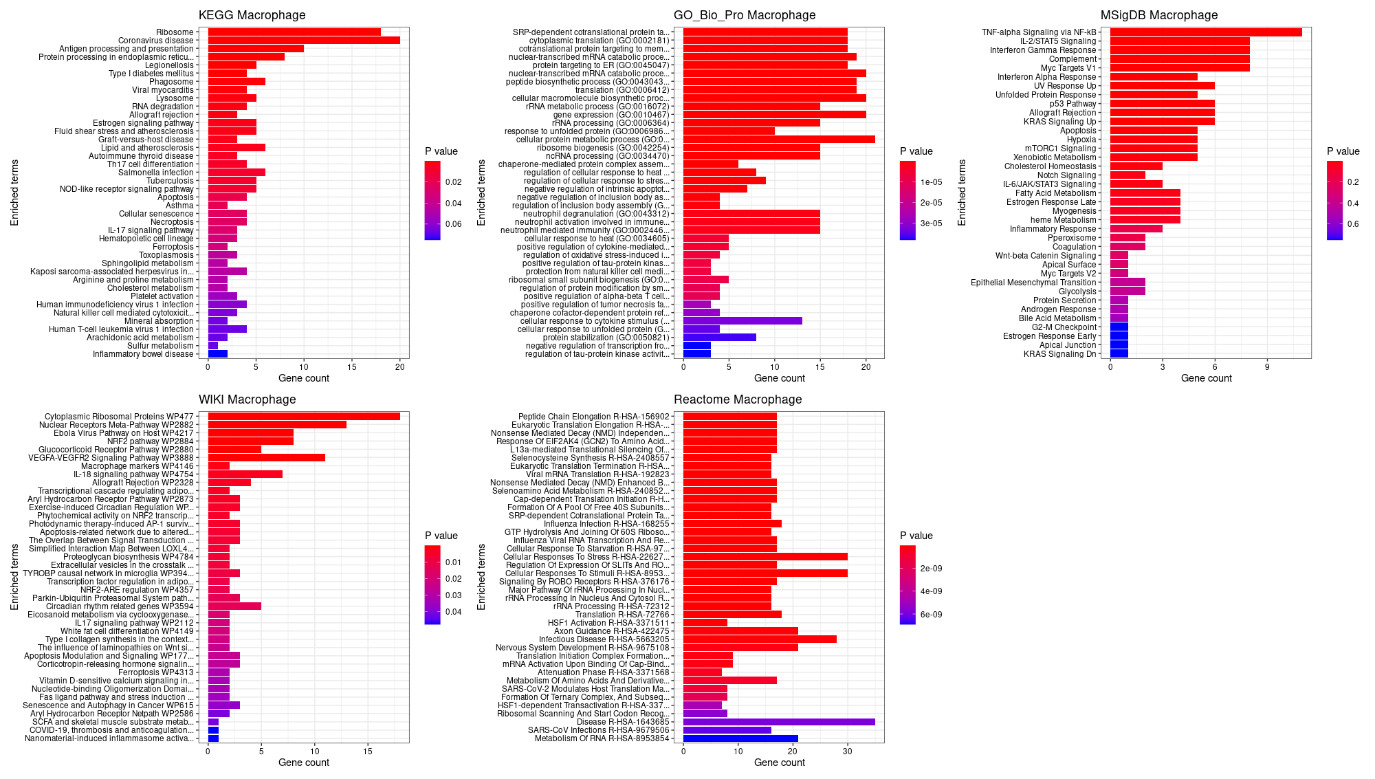
Fig H. Pathway enrichment analysis for COVID-19 brain macrophage cell types**

**Fig I. Pathway enrichment analysis for COVID-19 brain astrocyte cell types**

**
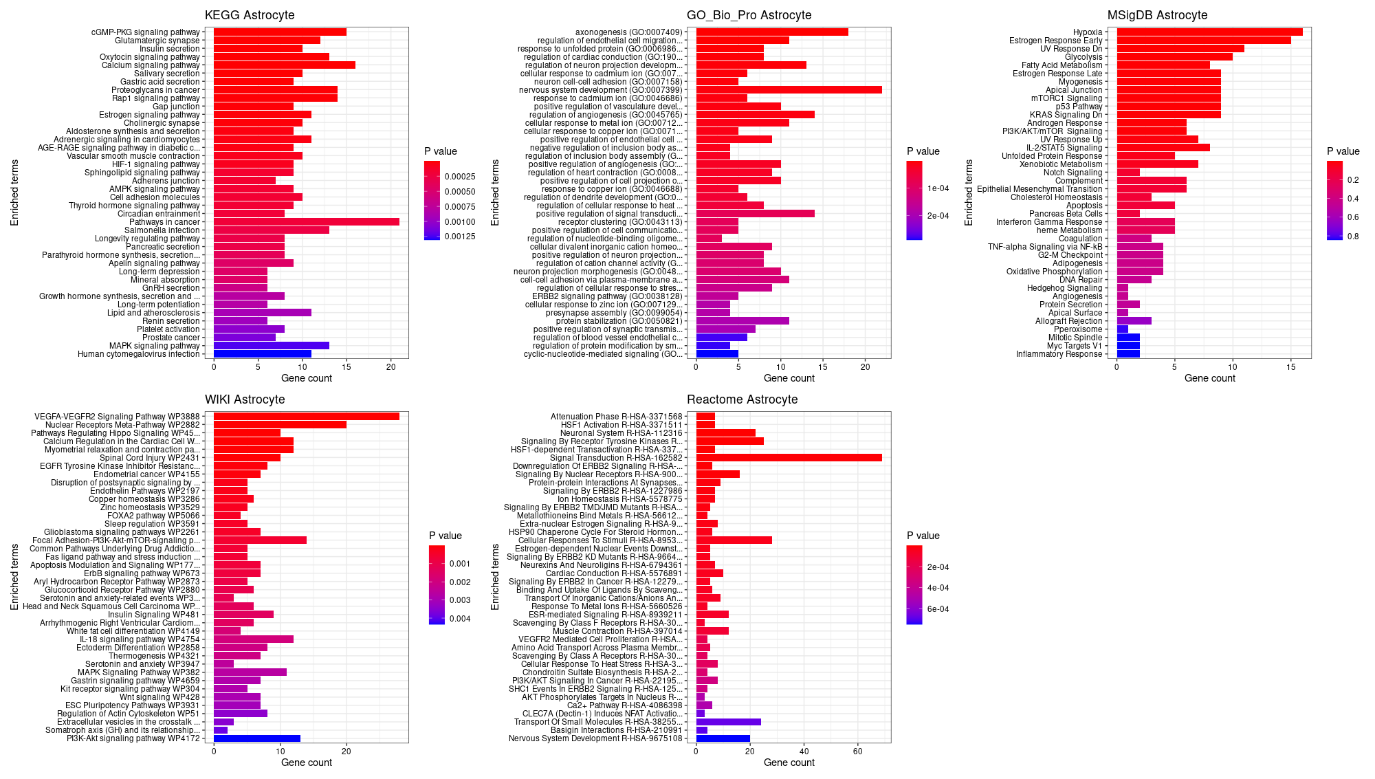
**

**Fig J. Pathway enrichment analysis for COVID-19 brain excitatory neuron cell types**

**
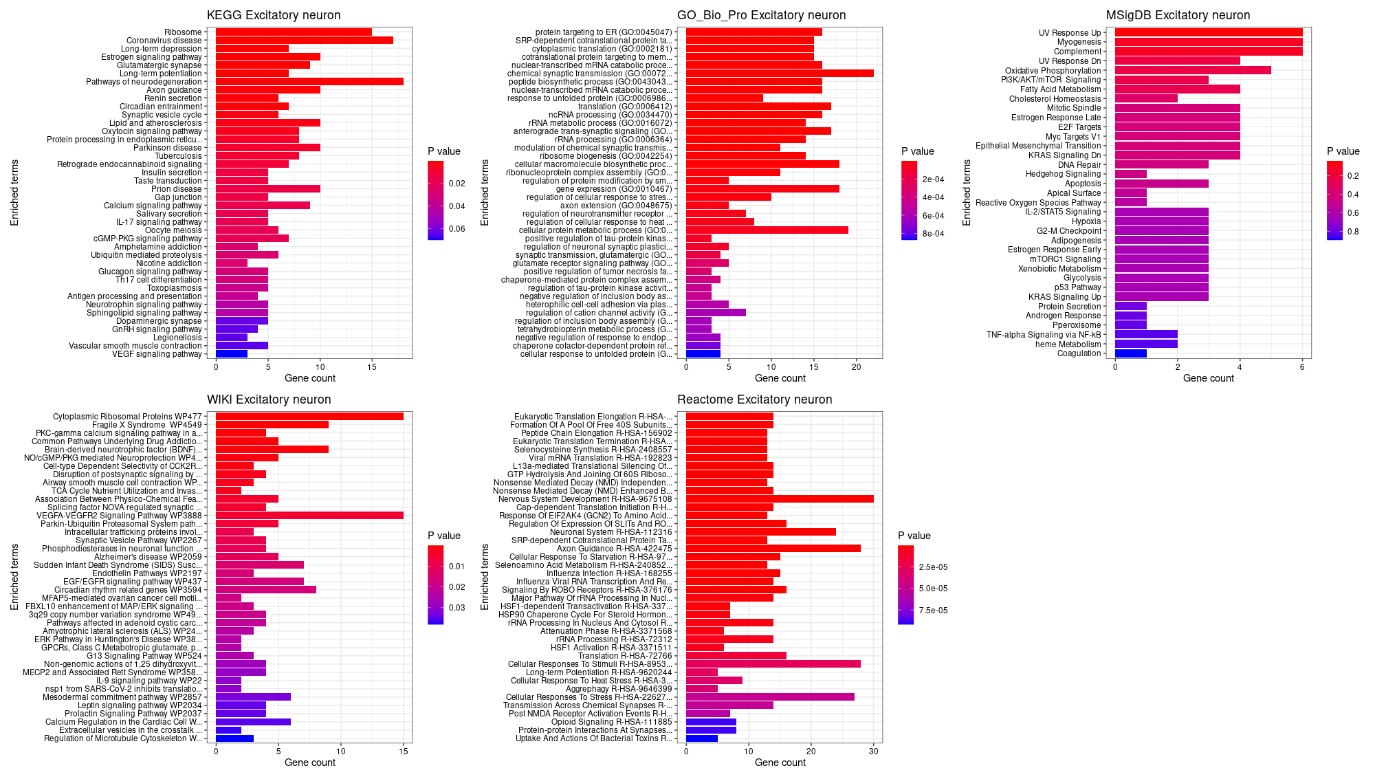
**

**Fig K. Drug repurposing via enrichR for COVID-19 brain excitatory neuron cell types**

**
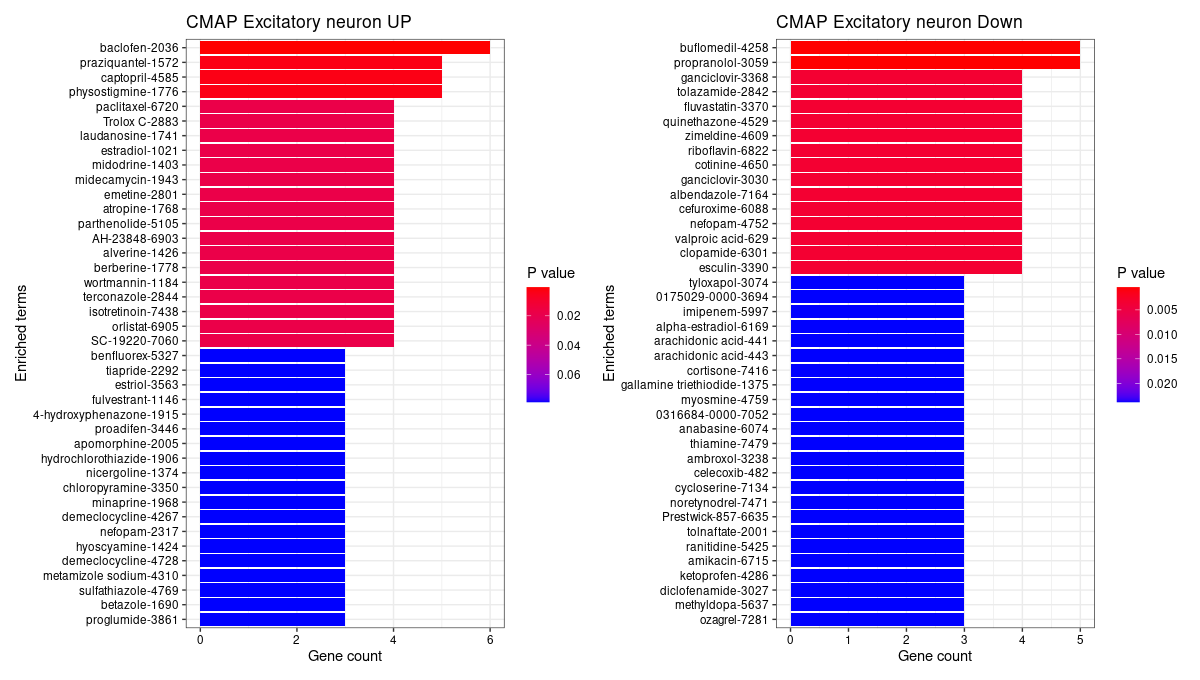
**
